# Prevalence of bullying by gender and education in a city with high violence and migration in Mexico

**DOI:** 10.26633/RPSP.2017.37

**Published:** 2017-04-14

**Authors:** Arnulfo Ramos-Jiménez, Rosa P Hernández-Torres, Miguel Murguía-Romero, Rafael Villalobos-Molina

**Affiliations:** 1 Instituto de Ciencias Biomédicas Universidad Autónoma de Ciudad Juárez, Ciudad Juárez Chihuahua Mexico Instituto de Ciencias Biomédicas, Universidad Autónoma de Ciudad Juárez, Ciudad Juárez, Chihuahua, Mexico.; 2 Facultad de Ciencias de la Cultura Física Universidad Autónoma de Chihuahua, Ciudad Juárez Chihuahua Mexico Facultad de Ciencias de la Cultura Física, Universidad Autónoma de Chihuahua, Ciudad Juárez, Chihuahua, Mexico.; 3 Facultad de Estudios Superiores Iztacala Universidad Nacional Autónoma de México TlalnepantlaMexico State Mexico Facultad de Estudios Superiores Iztacala, Universidad Nacional Autónoma de México, Tlalnepantla, Mexico State, Mexico.

**Keywords:** Bullying, aggression, violence, Mexico, Acoso escolar, agresión, violencia, México, Bullying, agressão, violência, México

## Abstract

**Objective.:**

To understand the prevalence of bullying, by gender and educational level, in Ciudad Juárez, Mexico, a city with high rates of violence and migration.

**Methods.:**

This was a cross-sectional, observational study conducted in 2012 – 2014 using a questionnaire known as the Bullying-Mexican. A probabilistic multistage cluster-sampling method obtained a study sample of 2 347 students (10 – 27 years of age) from the 400 000 enrolled in grade 5 – university level at the 611 public schools in Ciudad Juárez. Bullying prevalence and frequency (never, rarely, sometimes, often, every day) were analyzed with descriptive statistics. The statistical differences between males and females was assessed using a chi-square test; associations between frequency and academic level were determined by correspondence analysis and the Spearman Rho correlation. A multinomial logistic regression was performed to analyze whether gender and academic level acted independently in the frequency of bullying.

**Results.:**

Bullying prevalence was reported by 38% of females and 47% of males: ‘only victim’ represented 8.7%; ‘only aggressor,’ 13.2%; and ‘victim and aggressor,’ 21%. At higher levels of education, bullying prevalence declined; however, at the university, prevalence increased in the last semesters. Mockery and social exclusion were the two most dominant types of bullying, followed by beating, threats, and punishment.

**Conclusions.:**

The prevalence of bullying in Ciudad Juárez public schools is among the highest compared to other random studies and surveys. Bullying diminishes with age and educational level.

## RESUMEN

**Objetivo.:**

Comprender la prevalencia de intimidación o *bullying*, desglosada en función del sexo y el nivel de escolaridad, en Ciudad Juárez (México), ciudad con índices altos de violencia y migración.

**Métodos.:**

Este estudio transversal de observación se realizó entre el 2012 y el 2014 con el cuestionario mexicano sobre la intimidación (Bull-M). Tras aplicar el método probabilístico polietápico de muestreo por conglomerados, se obtuvo una muestra de 2 347 estudiantes (de 10 a 27 años) de los 400 000 matriculados entre el quinto grado y el nivel universitario en las 611 instituciones de educación pública de Ciudad Juárez. Se analizaron la prevalencia y la frecuencia de la intimidación (nunca, rara vez, a veces, a menudo, todos los días) mediante estadísticas descriptivas. Se evaluó la diferencia estadística entre los hombres y las mujeres con la prueba de la ji al cuadrado y se determinó la asociación entre la frecuencia y el nivel académico mediante el análisis de correspondencias y la correlación ro de Spearman (R). Se aplicó una regresión logística polinómica para analizar si el sexo y el nivel académico eran variables independientes respecto de la frecuencia de la intimidación.

**Resultados.:**

La intimidación tuvo una prevalencia del 38% en las mujeres y del 47% en los hombres: refirieron ser “solo víctimas” el 8,7%; “solo agresores” el 13,2%; y “víctimas y agresores” el 21%. La prevalencia de la intimidación disminuye a medida que aumenta el nivel de escolaridad, aunque se registró un incremento en los últimos semestres de la universidad. Los dos tipos más predominantes de intimidación son la burla y la exclusión social, a los que siguen la agresión física, la amenaza y el castigo.

**Conclusiones.:**

La prevalencia de intimidación en las instituciones de educación pública de Ciudad Juárez se encuentra entre las más altas cuando se la compara con otros estudios y encuestas de diseño aleatorizado. La intimidación disminuye a medida que avanza la edad y aumenta el nivel de escolaridad.

## RESUMO

**Objetivo.:**

Compreender a prevalência de *bullying*, estratificada por gênero e escolaridade, em Ciudad Juárez, México, um município com altos índices de violência e migração.

**Métodos:**

Estudo transversal observacional realizado em 2012-2014 usando o instrumento Bullying-Mexican. Utilizando um método probabilístico de amostragem multietápica por conglomerados, obteve-se uma amostra de 2 347 estudantes (10-27 anos de idade) do universo de 400 000 estudantes matriculados da 5^a^ série ao ensino superior nas 611 escolas públicas de Ciudad Juárez. A prevalência e frequência (nunca, raramente, às vezes, frequentemente, todos os dias) de *bullying* foram analisadas mediante estatísticas descritivas. A diferença estatística entre homens e mulheres foi avaliada através do teste qui-quadrado; associações entre frequência de ***bullying***e escolaridade foram determinadas mediante análise de correspondência e coeficiente de correlação de Spearman (R). Foi realizada análise de regressão logística multinomial para determinar se gênero e escolaridade influenciaram independentemente a frequência de *bullying*.

**Resultados.:**

O *bullying* foi relatado por 38% das mulheres e 47% dos homens: 8,7% identificaram-se como “somente vítimas”, 13,2% como “somente agressor”, e 21% como “vítima e agressor”. Nos níveis mais altos de escolaridade, a prevalência de *bullying* foi menor; porém, no ensino superior, aumentou novamente nos últimos semestres. Assédio verbal e exclusão social foram os dois tipos mais comuns de *bullying*, seguidos de agressão física, ameaças e castigos.

**Conclusões.:**

A prevalência de *bullying* nas escolas públicas de Ciudad Juárez está entre as mais elevadas já relatadas em estudos randomizados e levantamentos. O *bullying* diminui conforme a idade e escolaridade aumentam.

Bullying is characterized as repeated aggressive behavior exhibited as physical and/or psychological, systematic mistreatment between two parties (1).

Children who are victims of bullying, and aggressors (bullies) to a lesser degree, have higher incidences of health complaints, learning problems (2), depression, and long-term social problems in interpersonal relationships (3–5); these may result in domestic violence, criminality, substance abuse, and even suicidal thoughts (4, 6, 7).

Bullying occurs in nearly all social groups. Some of these groups are more susceptible to this problem and its consequences than others; among these groups, children and youth who have just initiated their studies at different educational levels are some of the most vulnerable, i.e., those who are perceived as weak, are short in stature, who have a different religion/skin color/ethnicity, immigrants, and those with disabilities, among others (8–11). Craig and colleagues (12), in their cross-national study on bullying and victimization in 40 countries (*n* = 202 056), showed that the prevalence of bullying in high schools ranged from 9%–45% and from 5%–36% among males and females, respectively. Verbal (~66%), physical (~50%), and social exclusion (~47%) are the most common representations (13, 14). The principals of these schools reported several causes of this phenomenon, such as psychological problems (56%) and social positioning (41%) for the bully, and being deviant, different, odd, or perceived as not fitting in (44%) for the bullied (14). All of these features are observed in populations with high migration and violence (11).

On the other hand, it has been reported that bullying prevalence may be aligned with educational level (9, 10), and that in large part, it is a learned behavior (9, 15) prevailing where violence, migration, and unsolved social problems exist (16). In this context, this study examines bullying prevalence among students from different educational levels in Ciudad Juárez, Chihuahua, in Mexico, a city with high violence and migration.

## MATERIALS AND METHODS

This was a cross-sectional, observational study conducted in 2012–2014 using a self-reporting questionnaire known as the Bullying-Mexican (Bull-M), previously validated among the study population (17).

### Participants

The study sample of 2 347 students from 10–27 years of age was representative of the metropolitan area of Ciudad Juárez. To obtain a random and appropriate sampling that included both genders and all academic grade levels, the study employed a probabilistic multistage cluster sampling method.

Participants were selected from the 400 000 students enrolled in grade 5–university level at all 611 public schools in Ciudad Juárez in 2012: 460 elementary schools, 106 junior highs, 33 high schools, and 12 universities (18). Equal numbers of male and female participants were chosen from each academic level and school grade (stratified). Students below grade 5 were excluded as being too young to fully understand the questions.

The sample was taken considering an alpha of 0.01 with 2.5% accuracy and a theoretical bullying prevalence of 50%, in the equation:

$$n=\frac{NZ_{\text{a}/2}^{2}P(1-P)}{\left(N-1){\text{e}}^{2}+Z_{\text{a}/2}^{2}P(1-P\right)}$$

Responder efficiency was 100%, i.e., if a student was not in the classroom at the time of the test, the next on the list occupied his/her place until the required sample size was obtained.

### Procedures

Students interviewed were those at school on the day that the Bull-M questionnaire was applied in the classroom by an interviewer (17). A total of 1 162 girls and 1 185 boys from four educational levels participated: 1 110 students from 17 elementary schools in grades 5–6 (10–11 years of age); 560 students from six junior high schools in grades 7–9 (12–15 years of age); 360 students from six high schools in grades 10–12 (15–18 years of age), and 317 students from one university in semesters 1–9 (19–27 years of age).

Bullying prevalence was assessed using the previously validated Bull-M test (17): Content validity = 0.93; Cronbach’s α = 0.75; Kaiser–Meyer–Olkin = 0.77. The test included 10 questions that employed a Likert-type scale, i.e., each item offered five options grouped in three factors (victim’s role: questions 1–5; aggressor’s role: questions 6–9; general health status: question 10). This questionnaire design enables respondents to anonymously record the prevalence, frequency, and type of bullying that the student might be facing or perpetrating.

Interviewers were trained in questionnaire application. Participants were asked to complete all of the questions in 20 minutes; no questionnaires were incomplete. The questionnaire was administered only when the interviewer was present. Groups comprised up to 30 students.

### Statistics

The prevalence and frequency of bullying (never, rarely, sometimes, often,every day) were analyzed with descriptive statistics. The “sometimes” frequency was used as a cut-off point to determine the prevalence of bullying among the students. The statistical difference between boys and girls was assessed using the chi-square test (χ^2^), while associations between frequency and academic level were determined by correspondence analysis and the Spearman Rho correlation (R). A multinomial logistic regression was performed to analyze whether gender and academic level acted independently in the frequency of bullying.

### Ethics

Written informed consent was obtained from the school administrators, the participants, and from their parents for those under 18 years of age. If consent was withheld by any student or school, an alternate was randomly selected from the stratus (academic level and grade). The protocol was approved by the schools’ authorities and by the Ethics Committee of the Universidad Autónoma de Ciudad Juárez (Chihuahua, Mexico).

## RESULTS

As shown in Table 1, about 38% of males and 47% of females reported participating in or suffering from bullying, with a prevalence of 43% that was higher in males (χ^2^ = 19.2; *P* < 0.001). In addition, both gender and academic level explained 5.9% in variance with respect to the prevalence of bullying (Nagelkerke Pseudo R2 = 0.059; *P* < 0.001). Male and female victims, including those who were also bullies, represented 29.7%; and male and female bullies, including those who were also victims, represented 34.2% of the total sample.

**TABLE 1.** Prevalence of bullying and victimization, by gender, among 2 347 public school students in grades 5 to university (10–27 years of age) in Ciudad Juárez, Mexico, 2012–2014

Bullying	Female		Male		Odds ratio	95% CI^a^	*P* value
	n	%	n	%			
Uninvolved	715	61.5	623	52.6			
Only victim	92	7.9	112	9.5	1.21	0.91–1.62	0.11
Only bully	143	12.3	167	14.1	1.17	0.92–1.49	0.11
Bully-victim	212	18.2	283	23.9	1.41	1.15–1.72	<0.01
Total bullying	447	38.4	562	47.5	1.44	1.22–1.70	< 0.01

***Source:*** Prepared by the authors from the study data.

a 95% Confidence Interval.

**TABLE 2.** Prevalence of involvement in bullying and victimization by academic level in students (n = 2 347; 1 162 females; 1 185 males) in Ciudad Juárez, Mexico, 2012–2014

	Elementary school		Junior high		High school		University		Chi-square test	*P* value
	n	%	n	%	n	%	n	%		
Uninvolved	525	47.3	332	59.3	253	70.3	228	71.9		
Only victim	111	10.0	55	9.8	22	6.1	16	5.0	16.0	<0.01
Only bully	134	12.1	81	14.5	47	13.1	48	15.1	37.3	<0.01
Bully and victim	340	30.6	92	16.4	38	10.6	25	7.9	91.2	<0.01

***Source:*** Prepared by the authors from the study data.

**TABLE 3.** Prevalence of involvement in bullying and victimization among university students (*n* = 317), Ciudad Juárez, Mexico, 2012 – 2014

Bullying	Semester								
	1st	2nd	3th	4th	5th	6th	7th	8th	R^a^
Uninvolved	86.7	77.1	91.3	69.8	80.0	70.6	22.2	50.0	-0.76^b^
Only victim	6.7	3.6	0.0	8.5	0.0	3.9	11.1	0.0	-0.10
Only bully	6.7	12.0	8.7	15.1	20.0	15.7	33.3	30.0	0.93^c^
Bully-victim	0.0	7.2	0.0	6.6	0.0	9.8	33.3	20.0	0.76^b^

***Source:*** Prepared by the authors from the study data.

a Spearman Rho correlation.

b *P* < 0.05

c *P* < 0.01

**TABLE 4.** Prevalence (%) of types of bullying in descending order, among 1 347 students (*n* = 1 347; 1 162 females and 1 185 males) from 10–27 years of age in the public schools of Ciudad Juárez, Mexico, 2012–2014

Types of bullying	Victims	Bullies
	%	%
Mocked	21.1	24.0
Excluded	15.7	20.2
Blamed	14.4	15.5
Rejected	11.8	15.4
Insulted	11.0	12.6
Accused	10.4	11.5
Forced	9.1	11.5
Expelled	8.6	10.5
Beaten	6.6	7.4
Hurt	6.4	6.7
Threatened	6.0	6.2
Punished	5.5	5.9

***Source:*** Prepared by the authors from the study data.

Prevalence was found to be different among academic levels (*P* < 0.001) (Table 2), observing a total decrease from 52.7% in elementary to 28% in university. Moreover, whereas victims diminished (R = -0.99; *P* < 0.001), bullies increased (R = 0.80; *P* < 0.01) according to academic level. However, among university students, the result was the inverse (R = 0.65 and 0.93), with a total increase from 13.3%–50% from the beginning to the end of the students’ university studies (Table 3).

The most prevalent types of bullying were mockery and social exclusion, while the least prevalent were threats and corporeal punishment (Table 4).

## DISCUSSION

This work investigates bullying prevalence and modifications among students of four educational levels in Ciudad Juárez, Mexico, a border city with high rates of violence, migration, criminality, and political and police corruption.In 2006, Ciudad Juárez was considered to be the most dangerous city in the world (19, 20).Coupled with corruption and the limitations of the local andfederal governments, children and youth become engaged in violence and criminal actions (19–21), which increase the prevalence of bullying (6, 9, 13). Considering that “rarely” reporting bullying is not bullying, we found a prevalence of 43%, including victims and bullies; this is among the highest prevalence rates reported by epidemiological surveys and probabilistic studies (12, 22–25). In this regard, high bullying prevalence has been reported in the United States (40%–71%) (14, 26), India (60%) (23), and Korea (40%) (24); moderate prevalence in the Netherlands(33%) (25); and lower prevalence in Brazil (8.5%) (27) and Taiwan (11%) (28).In the rest of Latin America, a report revealed a 17%–39% prevalence (29). In Mexico,the 2006 National Survey for Health and Nutrition found bullying prevalence to be 24.7% among youth 10–19 years of age (30); this increased to 30% in 2012 (31).Prevalence of bullying in Ciudad Juárez is much higher than it is in the rest of the country, suggesting urgent intervention programs are needed.

Because bullying gives rise to negative effects on an individual’s social health, several countries have studied this phenomenon in their populations (8, 23, 24, 28, 29). In addition, it is known that bullying increases in highly marginalized populations and highly violent zones, and appears to be associated with racial aspects (9, 25). In this sense, northern Mexico is an area of high migration and is a transport route for addictive substances into the United States; individuals failing to enter the United States often stay in the border zone (21), adding to the violence that is already higher here than in any other part of Mexico (32). Ciudad Juárez, as a border city, has high migration, and in the last decade, the dramatic increase in violence, could be affecting school health and bullying (6, 7, 11).

Bullying prevalence that is higher among boys than girls is a common phenomenon (33); however, we found a general decrease from 52.7% to 28.0%, which appears to be associated with academic grade, from elementary school to university. This result indicates that a diminishing prevalence of bullying is related to age and/or academic grade (33).In a similar manner, Brame and colleagues (34) found that bullying among children tends to diminish with age (from 6–13 years of age), but it increases and even becomes more violent during adolescence (14–16 years of age). On the other hand, Curwen and colleagues (35) reported that from high school to university, verbal and physical bullying diminishes, although it continues to occur in a passive, social form, such as mockery and rejection.

In contrast, our study found an increase in prevalence (from 13.3% to 50%), especially in bullies and bully-victims (from 0%–7% to 20%–30%), as well as in frequency (from “rarely” to “sometimes” and “often”) at the university level (data not shown). A possible explanation for this increase is that along with the challenges of co-existence at the university, issues of trust and difficulties/stress among students also take place (36, 37). In our studied population, mainly among university students, mockery, social exclusion, and blame were the most prevalent types of bullying; however, threats and punishments were the least prevalent types of bullying, and beating moved from 9th place among freshmen in the university to 3rd place among seniors (data not shown). These data indicate an increase in the violence level among these students, already observed in high schools (34), but opposite to that observed in other works (33, 35). On the other hand, Jansen and colleagues (25), when evaluating bullying in the Netherlands elementary schools, found a higher prevalence than ours, the order being bullies (17%), bullies-victims (13%), and only victims (4%).

The difference found in this study compared with the reported data may reflect several factors (38), among which may be the following:

- Methodological differences
  - validity and trust in the applied instruments
  - type of instrument applied (direct or indirect observation,questionnaire, interview, etc.)
  - complexity in instrument design and structured
  - form of question (open, closed)
  - bias in the questions
  - order and number of questions
- Design and site of instrument application
  - permitting anonymity or
  - not individual or grouped
  - classroom, schoolyard, or home
- Beliefs about violence
- Individual, social, and cultural differences
  - ethnicity
  - family socioeconomic status
  - gender, age, schooling
  - urban or rural settings
  - religion
- Differences due to time (because bullying and its perception can be modified over time).

The social problems and violence observed in the streets appear to affect healthy relationships among students, at least in this border city. This speaks to a possible reconsideration of school education in cities with similar characteristics, i.e., not only to promote knowledge, skills, and psychomotor development in schools, but also to strengthen humanitarian activities that foster empathy and a collaborative environment among students.

### Limitations

Because the structure of bullying is multifactorial and because questionnaires to detect it are subjective, we recommend applying more than one instrument to determine its prevalence, and then validating the convergence of information (39). In the current case, we evaluated presence, frequency, and type of bullying with only the Bull-M instrument. In addition, at the university level, we evaluated bullying in only one, the largest, university. Lastly, it could be important to apply the Bull-M in student populations across several cities to confirm or negate the assertions made in the present study.

### Conclusion

Bullying prevalence in Ciudad Juárez, Mexico, is among the highest reported to date. Prevalence and frequency were found to be slightly higher in male than in female students. Both prevalence and frequency of bullying diminished with age and academic grade; however, violence and social insecurity have affected the city’s school environment, making it aggressive and insecure. In the absence of programs that promote human values, school bullying is a natural consequence. It is necessary to implement programs that modify aggressive behaviors and strengthen effective communication and collaboration among students.

### Acknowledgements.

The authors wish to acknowledge the support and funding of the *Programa de Mejoramiento del Profesorado* of the Secretariat for Public Education (Mexico City, Mexico). Rafael Villalobos-Molina was a visiting professor at the Universidad Autónoma de Ciudad Juárez (Ciudad Juárez, Chihuahua, Mexico) and was supported in part by *Programa de Apoyos para la Superación del Personal Académico de la Universidad Nacional Autónoma de México* (Mexico City, Mexico) and Department of the Faculty at the Universidad Autónoma de Ciudad Juárez.

### Disclaimer.

Authors hold sole responsibility for the views expressed in the manuscript, which may not necessarily reflect the opinion or policy of the *RPSP/PAJPH* and/or PAHO.
